# The Role of CXCR3 in Nervous System-Related Diseases

**DOI:** 10.1155/2024/8347647

**Published:** 2024-10-11

**Authors:** Fangyuan Wang, Bing Guo, Ziyang Jia, Zhou Jing, Qingyi Wang, Minghe Li, Bingqi Lu, Wulong Liang, Weihua Hu, Xudong Fu

**Affiliations:** Department of Neurosurgery, The Fifth Affiliated Hospital of Zhengzhou University, Zhengzhou University, Zhengzhou, China

**Keywords:** G-protein receptor, inflammation, inflammatory chemokines, nervous system-related diseases, neurodegenerative diseases, pain

## Abstract

Inflammatory chemokines are a group of G-protein receptor ligands characterized by conserved cysteine residues, which can be divided into four main subfamilies: CC, CXC, XC, and CX3C. The C-X-C chemokine receptor (CXCR) 3 and its ligands, C-X-C chemokine ligands (CXCLs), are widely expressed in both the peripheral nervous system (PNS) and central nervous system (CNS). This comprehensive literature review aims to examine the functions and pathways of CXCR3 and its ligands in nervous system-related diseases. In summary, while the related pathways and the expression levels of CXCR3 and its ligands are varied among different cells in PNS and CNS, the MPAK pathway is the core via which CXCR3 exerts physiological functions. It is not only the core pathway of CXCR3 after activation but also participates in the expression of CXCR3 ligands in the nervous system. In addition, despite CXCR3 being a common inflammatory chemokine receptor, there is no consensus on its precise roles in various diseases. This uncertainty may be attributable to distinct inflammatory characteristics, that inflammation simultaneously possesses the dual properties of damage induction and repair facilitation.

## 1. Introduction

The nervous system consists of the central nervous system (CNS) and peripheral nervous system (PNS), with the former encompassing the brain, spinal cord, optic nerve, and retina, and the latter including all other nervous tissues such as the 11 pairs of cranial nerves (excluding the optic nerve), spinal nerves, autonomic PNS, and special sensory nervous system. There are notable differences between these two systems at both the tissue and cellular levels. For instance, myelinated axons in the PNS are coated with myelin formed by Schwann cells except for their initial and terminal segments, whereas myelin in the CNS is produced by coiled axons wrapped by flat membranes at the ends of oligodendrocyte processes. Additionally, the glial cell composition varies between the two systems. Glial cells in the CNS mainly include astrocytes, oligodendrocytes, microglia, ependymal cells, and radial glial cells. Astrocytes play roles in neurodegenerative diseases (NDDs) such as Parkinson's syndrome, prion infection, and autoimmune encephalomyelitis [1–3]; oligodendrocytes form the myelin sheath of the CNS; microglia is a specialized type of macrophage-derived from the mesoderm; ependymal cells play an important role in the nervous system early in human life [4]. PNS glial cells include Schwann cells, satellite cells, and enteric glial cells. Schwann cells form the myelin sheath around the peripheral nerves; enteric glial cells play roles in regulating the enteric nervous system and in parkinsonism [5, 6]. These distinctions underscore the need to separately consider the role of C-X-C chemokine receptor (CXCR) 3 in the CNS and PNS when studying nervous system-related diseases.

Neurological diseases are the leading cause of disability-adjusted life-years (corresponding to the sum of years of life lost and years lived with disability) and the second leading cause of death [7]. In addition to being seriously life-threatening, neurological diseases usually continuously impair normal physiological function, greatly impact daily life and mental health, and markedly reduce the quality of life. In addition, many patients with neurological diseases impose a huge burden on both their families and society as a whole due to an inability to live independently. Various factors such as mechanical injury, ischemia, hypoxia, anesthetics, infection, and self-hypersensitivity can damage the CNS and PNS. These pathogenic factors can lead to neuronal damage, separation of neurons and glial cells, structural and functional damage to the blood–nerve barrier, abnormal release of neurotransmitters, and abnormal electrical activity on axons. The repair ability of the nervous system is unfortunately extremely weak, and central neurons are completely incapable of repair [8], resulting in the available treatment programs often having little effect [9]. Therefore, there is an urgent need for innovative research aimed at improving clinical treatments for nervous system diseases. Besides neurological diseases, nervous tissue plays crucial roles in many systemic diseases, making the exploration of the nervous system's roles in disease a vibrant area of medical research.

Injury to the CNS and PNS can result in the release of various cytokines, including neurotransmitters, lipid mediators, neurotrophic factors, and chemokines [10], which play key roles in the development and prognosis of diseases. Among them, inflammatory chemokines mediate multiple key relationships in nerve injury and repair. This has led to a growing interest in the role of chemokines in the nervous system and diseases over the past decade.

Inflammatory chemokines are a group of G-protein receptor ligands with conserved cysteine residues [11, 12] divided into four main subfamilies: CC, CXC, XC, and CX3C. The CXC family contains 17 members (CXCL1–CXCL17) with 7 receptors (CXCR1–CXCR7). The ligands of CXCR1 include CXCL1, CXCL5, CXCL6, and CXCL8 [13–15], those of CXCR2 include CXCL1, CXCL2, CXCL3, CXCL5, CXCL6, CXCL7, and CXCL8 [16–18], and those of CXCR3 include CXCL4, CXCL9, CXCL10, CXCL11, and CXCL13. The ligand of CXCR5 is CXCL13 [19], that of CXCR6 is CXCL16 [20], and that of CXCR4 and CXCR7 is CXCL12 [21]. Previous studies have suggested that members of the CXC family are widely involved in the pathological processes underlying nervous system-related diseases, among which the CXCR3 receptor is widely expressed in various cells in the CNS and PNS and plays roles in various diseases. This article explores the relationships of CXCR3 in the CNS and PNS with various diseases and provides abundant theoretical evidence for research into nervous system diseases.

## 2. CXCR3 Background

CXCR3 is a common G-protein-coupled receptor that is mainly distributed in various tissues such as the adrenal gland, bone marrow, lymph nodes, and brain [22, 23]. The CXCL4, CXCL9, CXCL10, CXCL11, and CXCL13 ligands of CXCR3 [24–26] have different affinities for this receptor [27, 28], and CXCL13 can also bind to CXCR5 [25, 29]. The ligands of CXCR3 are mainly distributed in immune cell lines, but they are also expressed in certain neuronal cell lines. CXCL4 can be expressed in platelets and activated microglia cells [30, 31]; CXCL9 and CXCL10 are expressed in fibroblasts, endothelial cells, and dendritic cells [32–34]; CXLC10 can also be expressed in Schwann cells [35]; and CXLC11 is primarily expressed in fibroblasts, glial cells, and endothelial cells [36–38]. It is noteworthy that the functions of both CXCR3 and its ligands are interdependent, since CXCR3 exerts its physiological effects through binding to its ligands and subsequently activating downstream signaling pathways.

The CXCR3 pathway plays a pivotal role in various lesions in the CNS and PNS. The CXCR3 pathway in the CNS plays a role in diseases, including glioblastoma, traumatic optic neuropathy (TON), neuropathic pain, and CNS degenerative diseases such as Alzheimer's disease (AD), multiple sclerosis (MS), prion diseases (PrD), and bipolar disorder (BD). However, there has been insufficient research into the role of CXCR3 in the PNS. Most studies have only focused on neurofibromas, neural tissue in tumor microenvironments (TMEs) from nonneural-origin tumors, and neuropathic pain. Although neuropathic pain is associated with pathological changes in both the CNS and PNS, most research into neuropathic pain relies on various animal models of peripheral neuroinflammation, which are referred to here as PNS-related diseases. In summary, research into CXCR3 and also other CXC-family receptors in the nervous system mainly focuses on CNS-related diseases, while research into PNS-related diseases mainly focuses on pain. The pathways of CXCR3 and its ligands in the nervous system are summarized in Table 1, and the detailed discussion below will focus on their functions and mechanisms in diseases related to the nervous system.

## 3. Role of the CXCR3 Pathway in CNS-Related Diseases

### 3.1. Glioma

Gliomas originate from glial cells, and CXCR3 and its ligands are widely expressed in various glial cells. This situation makes it crucial to pay attention to the role of CXCR3 and its ligands in glioma. Previous research has indicated that CXCR3 contributes to glioma invasiveness, tumor immune responses, and antitumor drug treatments.

CXCR3 and its ligands play important roles in the invasion and migration of glioma. Pu et al. [54] suggested that strong expression of CXCR3 is an independent factor for a poor prognosis of glioma and high tumor invasiveness. This may be due to significant increases in the calcium flux [45] and ERK1/2 [45, 46] phosphorylation mediated by the CXCL10/CXCR3 pathway, which enhances tumor invasiveness. Furthermore, the effects of CXCR3 are influenced by internalization in addition to being regulated at the transcription and translation levels. Boyé et al. [45] suggested that clathrin is a key factor in CXCR3 internalization. Lipoprotein receptor-related protein-1 (LRP1) enhances the internalization of CXCR3 by regulating clathrin and reduces the number of CXCR3 on the cell membrane and the invasiveness of glioblastoma, and it is often downregulated in the invasive region. The adverse effects of CXCR3 have also been verified in mechanistic studies of the antitumor drug celecoxib. The level of cyclooxygenase (COX)-2 in the human body is often increased by various stimuli, and this has the ability to increase the malignant potential of glioma [55]. This means that celecoxib (a selective inhibitor of COX-2) can exert antitumor effects. In addition to the COX-2-dependent pathway, celecoxib was able to directly inhibit and induce apoptosis of low-grade gliomas via the Akt/surviving and Akt/ID3 pathways, respectively [56]. Based on this, Shono et al. [47] investigated the antitumor mechanism of celecoxib based on a mouse glioma model. They found that although celecoxib did not affect CXCL10, it significantly reduced the expression of CXCR3 via the NF-*κ*B pathway, thereby blocking the CXCL10/CXCR3 pathway to exert an antitumor effect. All of these findings demonstrate that CXCR3 can promote tumor growth and migration and has a negative effect on prognosis.

In the context of glioma immunotherapy, the prevailing consensus is that activation of the CXCR3 pathway can enhance peritumoral T-cell infiltration and subsequently exert tumor-suppressive effects. Based on an investigation of a mouse glioblastoma model, Zhao et al. [57] suggested that T cells expressing fibrinogen-like-2-specific single-chain variable fragments can increase the number of tumor-specific CD69^+^CD8^+^ memory T cells by binding to CXCL9, CXCL10, and CXCR3, as well as activating downstream pathways. Cheever [58] observed similar results when exploring the mechanism of poly(I:C) promoting immunotherapy. Poly(I:C) is considered an immunostimulant with great potential in cancer immunotherapy, and those authors suggested that it acts by inducing type-I IFN (interferon) secretion in primary glioblastoma via autocrine or paracrine pathways. Activation of the IFN-*α* and IFN-*β* receptor subunit 1 IFNAR can lead to tumor-cell-mediated CXCL10 release [48]. CXCL10 will recruit CD8^+^ T cells to infiltrate around glioma lesions and activate the immune response of glioma. However, Fu et al. [59] found that although CXCR3 ligands recruited large numbers of NK cells exhibiting strong CXCR3 expression, these cells showed low IFN-*γ* expression levels and did not have the ability to dissolve cancer cells to exert tumor immune responses.

The previous findings indicate that CXCR3 exerts two opposing effects in glioma: (1) it promotes the dedifferentiation, migration, and invasion of tumors and recruits NK cells without a cytolytic effect, which seriously affects the prognosis of glioma patients; (2) CXCL9/CXCL10 secreted by tumors can recruit CXCR3-expressing T cells and enhance tumor immune responses. This duality makes CXCR3 as an effective target for glioma, and so the underlying mechanisms need to be further explored to identify suitable specific targets from its downstream pathway.

### 3.2. NDDs

NDDs are a group of diseases that gradually reduce the number of neurons and result in gradual losses in the structure and function of neural networks [60, 61]. Neuroinflammation is an important pathological marker of NDDs, especially microglia and astrocytes, which have important regulatory significance for NDDs. Previous studies have confirmed that CXCR3 and its ligands are widely expressed in microglia and astrocytes, and both have been confirmed to regulate demyelinating lesions. The endogenous activation of glial cells is a key step in cuprizone-mediated demyelination [62], and hence, the roles of CXCR3 and its ligands in NDDs are worth exploring further.

#### 3.2.1. AD

AD is a common degenerative disease of the CNS. It is generally believed that the core mechanisms underlying AD are the gradual deposition of extracellular *β*-amyloid (A*β*) and the accumulation of intracellular tau protein. Among them, the A*β* protein acts together with microglia to play an important pathological role in AD. First, it has been suggested that microglia, with continuous exposure to A*β*, will become self-invasive and produce neurotoxins, leading to AD [63]. However, studies have also shown that microglia can release A*β* hydrolases and express scavenger receptors, which mediate the phagocytosis of A*β* [64–66], and this phagocytosis process is regulated by the CXCR3 signaling pathway. Previous studies have shown that CXCL10 concentrations in cerebrospinal fluid (CSF) are positively correlated with the severity of cognitive impairment in AD patients [67, 68], while CXCR3-deficient mice exhibit significant reductions in plaque burden and A*β* levels [69], suggesting that the activation of CXCR3 pathways has negative implications for AD. It has also been shown that CXCL10 is expressed in astrocytes in AD [42], and CXCL10 was detected in close proximity to A*β* plaques in an AD mouse model [70]. CXCL10 secreted by adjacent astrocytes can act on CXCR3 in various cells and play a pathological role in three ways: (1) CXCL10 acting on CXCR3 in central neurons to activate the ERK1/2 pathway and induce the generation of amyloid deposits [42], (2) CXCL10 acting on CXCR3 on the membrane of T cells to regulate the aggregation of T cells to AD lesions and induce neuronal toxicity [71], and (3) CXCL10 produced directly or indirectly by fibrillar A*β* (fA*β*) can bind to CXCR3 receptors on the membrane of microglia and reduce the phagocytosis of fA*β* by microglia [69]. In addition to fA*β*, transactivation-response region DNA-binding protein 43 (TDP-43) is known to increase the expression levels of CXCL9 and CXCL10 by astrocytes in the hippocampus. Studies have shown the abnormal TDP-43 accumulation in hippocampal astrocytes in a mouse model of AD, which can indirectly effect on CXCR3 at hippocampal excitatory presynaptic terminals to promote neuronal overactivation and memory loss, resulting in cognitive decline in dementia patients [49].

In summary, the CXCL9, CXCL10 pathway is mainly produced by astrocytes and acts on CXCR3 on various cells, inducing a series of negative effects, and blocking this pathway can produce positive effects. Some small molecule antagonists of CXCR3 have the potential to be the new AD drugs and deserve further exploration.

#### 3.2.2. MS

MS is a demyelinating disease of the CNS characterized by sporadic patchy demyelination in the brain and spinal cord [72]. It is believed that the mechanism underlying MS is related to immunity, and various immune cells (e.g., lymphocytes) play important roles in the course of MS. However, viral infection (e.g., EBV) is also considered a key factor in its pathogenesis. Studies performed within the past 5 years have confirmed that CXCR3 plays roles in both of the above mechanisms. Moreover, examinations of the CSF of MS patients have revealed significant increases in CXCL10 concentrations [73, 74].

T cells are a key part of MS lesions. Various subtypes of CXCR3^+^ T cells play important roles in different stages of MS and in different treatment regimens. A clinical trial has shown that CD3^+^CD8^+^CXCR3^+^ cells were the only cell type with a significant association with T2-weighted lesions in patients with relapsing-remitting MS, which were strongly associated with longer duration of demyelination and axonal damage [75]. Ocrelizumab (OCRE), a recombinant humanized monoclonal antibody, selectively targets CD20-expressing cells [76–78]. Mathias et al. [79] found that OCRE can deplete CD8^+^CD20^+^ T cells and reduce their expression levels of CXCR3 and CNS-related lymphocyte function-associated antigen 1 (LFA-1) integrin to inhibit the development of MS. Previous studies have also found that the concentration of CXCL10 in the CSF increases to recruit T cells into the CSF during MS [73]. The higher levels of CXCL10 may be produced by astrocytes surrounding the lesion. Activated astrocytes are able to secrete CXCL9 and CXCL10 and induce T-bet production by activated CD4^+^ T cells, thereby amplifying the Th1 (T helper 1)/Tc1 (T cytotoxic 1)-like phenotype [80]. CD4^+^ T cells are the key cells involved in the acquired immune response. Naive CD4^+^ T cells can differentiate into different cell subsets including Th1, Th2, Th17, and regulatory T (Treg) cells, which play key roles in MS. Several studies have shown that blocking the differentiation of CXCR3^+^CD4^+^ T cells or inhibiting its differentiated cell subsets can be effective in treating MS. Diebold et al. [81] demonstrated that dimethyl fumarate provides immunomodulatory therapy for MS by depleting memory Th cells expressing GM-CSF, IFN-*γ*, and CXCR3. RING finger (RNF) proteins contain the RING domain and constitute the largest known E3 ubiquitin ligase family [82]. Wang et al. [83] found that RNF157 promotes histone deacetylase 1 ubiquitination and degradation based on the mouse experimental autoimmune encephalomyelitis (EAE) model. Inhibiting the differentiation of CD4^+^ T cells to Th17 and the expression of CXCR3 may slow the development of MS. Meanwhile, in smokers, GPR15 and CXCR3 are strongly expressed in CD4^+^ T cells, and the expression levels of GPR15 and CXCR3 are strongly correlated with the degrees of myelin degradation and functional impairment [84].

CD4^+^ T cells not only play a direct role in the development of MS but also promote the development of MS via B cells. Bogers et al. [85] demonstrated that brain-resident CD4^+^ memory T cells were able to mediate the conversion of CXCR3^+^ B cells to CD27^+^CD38^+^ antibody-secreting cells and to secrete IgG into the CSF. These antibodies may promote the development of MS via indirect mechanisms such as immune complex formation.

B cells also play an important role in MS. Van Langelaar et al. [86] suggested that the levels of CXCR3^+^ B cells in the CNS are higher in MS patients. Natalizumab enhances the ability of subsets of CXCR3^+^IgG^+^ cells to cross the blood–brain barrier. In addition, IFN-*γ* and TLR9 can induce the expression of T-bet and CXCR3 in MS patients to further enhance CXCR3-mediated recruitment to lesions and to trigger local lesions in MS patients. In contrast to IFN-*γ* and TLR9, evobrutinib, a Bruton's tyrosine kinase inhibitor, can block the conversion of naive B cells to CXCR3+ B cells and inhibit the CXCL10-mediated migration of memory B cells across the blood–brain barrier [87]. In summary, CXCR3+ B cells may play a pathogenic role in the development of MS.

CXCR3 and its ligands in the peripheral immune system also have a regulatory effect on MS. Xie et al. [88], based on their study using the mouse EAE model, suggested that methyl acetate treatment can elevate the expression levels of CXCL10 and CXCL9 in the spleen, as well as CXCR3 in Th1 cells. This treatment induces the retention of Th1 cells in the peripheral immune system, reduces their infiltration into the CNS, and may alleviate MS lesions.

EBV infection has been proposed as another important factor in the pathogenesis of MS. SoRelle et al. [89] suggested that a new EBV infection can induce a rapid increase in CXCR3+CD11c+FCRL4+ B cells, which are T-cell independent and can directly invade the nervous system to participate in the pathological processes of MS. Similar observations were made by Van Langelaar et al. [90], who demonstrated that EBV infection enhanced the transformation of CXCR3+ memory B cells into plasma cells, which significantly enhanced the ability of these plasma cells to secrete anti-EBNA1 IgG and triggered local lesions in MS patients. In addition, Leffler et al. [91] showed that CD4^+^ T cells activate monocytes to express CXCR3 and systematically migrate into the CNS after EBV infection by secreting IFN-*γ*. CXCR3^+^ monocytes that enter the CNS are able to activate CD8^+^ T cells and mediate cytotoxicity against glial cells and myelin.

#### 3.2.3. BD

BD is a mental illness characterized by alternating episodes of mania and depression [92]. Brietzke et al. [93] found that serum CXCL10 levels were significantly increased in BD patients. This observation was verified by Barbosa et al. [94] who examined the plasma levels of chemokines in BD patients in different mental states and found that CXCL10 and CXCL11 were significantly increased. Poletti et al. [95] further explored the relationship between serum inflammatory factors and cognitive ability and suggested that a low-grade CXCL10 state could contribute to neurocognitive deficits in BD patients. Studies of specific immune cells of BD patients have found that the percentage of CD4+CXCR3+ lymphocytes is decreased in BD patients. The proportion of T cells was also associated with BD. In both patients and healthy controls, the percentage of circulating Th17 (CCR6+CXCR3–CCR4+CCR10–) cells was positively correlated with higher fractional anisotropy in fiber tracts that promote brain functional integrity. However, the number of circulating Treg (CD4+CD25+FOXP3+) cells was positively correlated with higher radial and mean diffusivity values in patients [96]. This suggests that CXCR3+ T cells play a negative role in BD. BD is an immune-related central NDD, and previous studies have confirmed that CXCR3+ T cells play pathogenic roles in various NDDs. Future studies should, therefore, explore the role of CXCR3+ T cells in BD.

#### 3.2.4. PrD

PrDs are a group of infectious and fatal NDDs. Riemer et al. [2] suggested that inhibiting CXCR3 can inhibit the activation and migration of microglia and promote the proliferation of GFPA^+^ astrocytes to prolong patient survival after prion infection. Song et al. [97] found that brain tissue damaged after prion infection could recruit mesenchymal stem cells via the CXCR3 pathway to promote the repair of this tissue. This opens up the possibility of using mesenchymal stem cells to treat PrDs in the future.

### 3.3. TON

The optic nerve is 1 of the 12 pairs of cranial nerves and is considered part of the CNS since it originates from the optic canal during embryonic development, and its anatomical structure is consistent with that of other central nerves. TON involves the axonal degeneration of retinal ganglion cells (RGCs) caused directly or indirectly by optic nerve injury, leading to the death of RGCs and irreversible visual impairment [98]. Strictly speaking, TON is a special type of traumatic central NDD. Ha et al. explored the mechanism of RGC death after optic nerve injury based on a mouse model of optic nerve crush (ONC) and found that axonal injury caused a significant increase in CXCL10 expression in RGCs both in vitro and in vivo. CXCL10 will recruit and activate immune cells via the CXCL10/CXCR3 pathway and indirectly cause RGC damage while treating RGCs with CXCL10 alone in vitro also directly causes RGC apoptosis. Ha et al. also investigated the CXCL10 promoter region containing transcription factor binding sites and found that the phosphorylation of STAT1 and STAT3 was significantly increased in the early stage after ONC and that blocking this phosphorylation significantly inhibited the TON-mediated expression of CXCL10.

In summary, in the early stage after ONC, the expression of CXCL10 in RGCs is increased through the activation of STAT1 and STAT3. CXCL10 induces the degeneration of RGC axons by indirectly recruiting and activating immune cells and directly inducing RGC apoptosis, leading to TON [41]. However, the roles of the recruitment and activation of immune cells in TON remain controversial. Liu et al. [99] explored the role of the CXCL5/CXCR2 pathway in TON and believed that CXCL5 would promote the survival rate and axonal regeneration of RGCs after optic nerve injury and that this pathway could activate CD68^+^ cells to protect RGCs after lens injury. We, therefore, hypothesize that different CXC receptors and their downstream pathways play different roles in TON and that different immune cells play different roles in the development of this condition.

### 3.4. CXCR3 Pathway in the CNS

**Figure 1:**
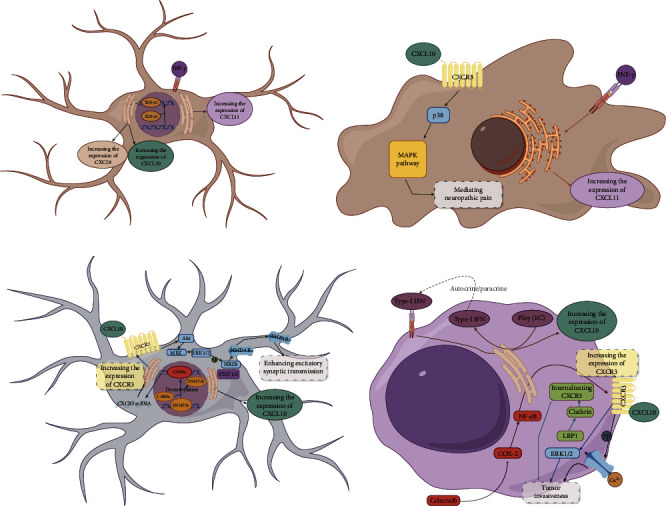
The pathway of CXCR3 and its ligands in CNS cells (by Figdraw). (A) Astrocyte: TDP-43 promotes CXCL9 and CXCL10 expression, and IFN-*γ* increases CXCL11 expression. (B) Microglia: IFN-*γ* activates its receptor to promote CXCL11 expression. CXCL10 activates the MAPK pathway via p38. (C) CNS neuron: phosphorylation of STAT1 and STAT3 promotes CXCL10 expression. C/EBP*α* can inhibit DNMT3b and the methylation of CXCR3 to reduce its expression. The binding of CXCL10 to CXCR3 can activate the downstream Akt/MEK/ERK1/2 pathway and then phosphorylate NR2B to enhance the activity of NMDAR and AMPAR. (D) Glioma: poly(I:C) can promote type-I IFN autocrine and paracrine pathways and act on glioma.

In the CNS, the CXCR3 signaling pathway exerts significant effects on various cell types, including astrocytes, microglia, neurons, and glioma cells, as depicted in Figure 1.

Astrocytes are primary producers of CXCR3 ligands such as CXCL9, CXCL10, and CXCL11. In conditions like AD, TDP-43 enhances CXCL9 and CXCL10 expression in astrocytes near lesions, leading to neuronal overactivation and memory impairment [49]. Furthermore, IFN-*γ* stimulates CXCL11 expression in both astrocytes and microglia through its receptor activation [27, 50].

In CNS neurons, both CXCL10 and CXCR3 are expressed, and their activation can trigger signaling cascades such as the MAPK pathway, notably p38, which regulates neuropathic pain in conditions like chronic constriction injury (CCI) [51]. Phosphorylation of STAT1 and STAT3 increases CXCL10 expression in RGCs, promoting the degeneration of RGC axons by indirectly recruiting and activating immune cells and directly inducing RGC apoptosis [41]. The expression of CXCR3 in CNS neurons is regulated by DNMT3b, a key protein maintaining CXCR3 methylation. Leucine zipper CCAAT-enhancer binding protein *α* (C/EBP*α*) inhibits DNMT3b, reducing CXCR3 expression [39]. Additionally, CXCL10 activation of CXCR3 in neurons leads to downstream effects on the Akt/MEK/ERK1/2 pathway [39, 40, 42]. This activation of ERK1/2 phosphorylates NR2B, enhancing synaptic transmission by modulating NMDAR and AMPAR activities and contributing to neuropathic pain mechanisms [39, 43, 44].

In gliomas, CXCL10 expression is mainly regulated by the type-I IFN pathway, particularly influenced by poly(I)-induced IFN autocrine and paracrine signaling through IFNAR1 activation [48]. Glioma cells also express CXCR3, regulated by pathways like COX-2/NF-*κ*B and LRP1-mediated internalization, which enhance tumor invasiveness and aggressiveness through ERK1/2 phosphorylation and calcium channel activation [45, 47].

Overall, the CXCR3 pathway in the CNS plays pivotal roles through multiple signaling pathways. Astrocytes are key producers of CXCR3 ligands, and both astrocytes and microglia respond to IFN-*γ* to regulate CXCL11 expression. The MAPK pathway, particularly p38 activation, emerges as a critical downstream mediator of CXCR3 signaling in neurological conditions, highlighting its physiological importance in CNS function and pathology.

## 4. Role of the CXCR3 Pathway in PNS-Related Diseases

There have been far fewer studies of CXCR3 in the PNS than in the CNS, with previous PNS-related studies only investigating the role of CXCR3 in neurofibroma, nonneurogenic tumors, and neuroinflammatory pain models based on peripheral neuropathy.

### 4.1. Neurofibroma

Neurofibroma is a heterogeneous benign peripheral nerve tumor that mainly originates from the PNS and is composed of various cells, including Schwann cells and fibroblasts [100]. There are two main neurofibroma phenotypes: plexiform neurofibromas (pNFs) and dermal or cutaneous neurofibromas. pNFs have malignant potential, and about 50% of cases of neurofibromatosis type 1 (NF1) are transformed from pNFs [101], which has attracted wide attention. However, the diffuse infiltration of plexiform fibroma often results in poor clinical effects of surgical treatment, which makes drug treatments to block the malignant transformation of the tumor particularly important. NF1 mainly encodes neurofibromin, which can inhibit the expression of the Ras gene and function as a proto-oncogene [102]. Previous studies have shown that biallelic deletion of the NF1 gene in Schwann cells is closely related to the pathogenesis of sporadic pNF [103, 104]. Fletcher et al. [53] found that the CXCL10/CXCR3 pathway played a decisive role in the stage of tumor formation in the NF1 mouse model of neurofibroma. CXCL10 was found to be specifically upregulated in Nf1 mutant mice, which might be produced by immature or even dedifferentiated Schwann cells after 2 months in Dhh-Cre Nf1fl/fl dorsal root ganglia (DRG), and its expression was associated with the Ras/MEK/ERK pathway. Meanwhile, large numbers of CXCR3-expressing T cells and dendritic cells were abnormally aggregated in Dhh-Cre Nf1fl/fl DRG. In the neurofibroma TME, Schwann cells recruit T cells and dendritic cells to the sites of neuroinflammation and neurofibroma lesions via the CXCL10/CXCR3 pathway. These immune cells directly or indirectly play important roles in tumor initiation and in the processes of Remak bundle disruption, nerve fibrosis, and sustaining macrophage accumulation in neurofibromas.

### 4.2. Nonneurogenic Tumors in the PNS

According to the WHO, cancer is now the leading cause of death in all countries worldwide [105]. Tumor tissue is a complex system. In addition to tumor cells, it includes various cells, such as immune cells and neurons, which together constitute the TME. It was once widely believed that the TME did not play an important role in tumor development. However, the increasing knowledge gained through cancer research has gradually led to the realization that cancer is a complex systemic disease and that the TME plays an important role in tumor receptors, while CXCR3 also has a unique role in the TME.

It has been confirmed that nervous tissues in the TME can play important roles both in the proliferation and migration of tumors and in cancer pain [106]. In a mouse breast cancer model, Hirth et al. [107] co-cultured the K8484 cell line of the PDAC mouse model with mouse DRG neurons in vitro and found that the amount of tumor cell migration was positively correlated with both the number of co-cultured DRG neurons and the co-culture time. However, the knockdown of CXCL10 and CXCL12 by siRNA significantly reduced cell migration. This suggests that neurons in the TME of breast cancer are able to promote tumor cell migration by releasing CXCL10 or CXCL12 and combining with CXCR3. Combined with the detection of downstream signaling proteins, those authors suggested that this promoting effect on tumor cell infiltration into peripheral nervous tissues is achieved by activating the downstream Akt, MEK, and RAC signaling pathways, among which Akt is a key pathway downstream of CXCR3 and so also plays an important role in cancer pain. Guan et al. [40] found that the expression levels of CXCL10 and CXCR3 in the rat spinal cord were significantly increased in a rat pain model of bone metastasis in breast cancer and that blocking the CXCL10/CXCR3 pathway could inhibit the activation of microglia and the development of cancer pain. Further experiments showed that CXCR3 was widely expressed in various neurons in the DRG and spinal cord, and CXCR3 was strongly positively correlated with the localization and expression of Akt and ERK1/2, suggesting that CXCR3 in the spinal cord mediates cancer pain after breast cancer bone metastasis via the Akt or ERK1/2 pathway.

In summary, the CXCL10/CXCR3/Akt pathway plays an important role in relieving breast cancer pain both in the early stage by inhibiting nerve metastasis and in the late stage of metastasis. This pathway is, therefore, a potential new therapeutic target that deserves more attention in studies of breast cancer.

### 4.3. Neuropathic Pain

Neuropathic pain is a refractory chronic inflammatory pain with an incidence of 7%–10% in the general population. Slow progress in the clinical translation of experimental drugs and novel treatment options has severely restricted the control of the disease using existing treatment options. Injury to either the central or peripheral somatosensory system can cause neuropathic pain, but the experimental surgical options for the PNS (especially the sciatic nerve) are more convenient and effective. Therefore, most current research on neuropathic pain utilizes CCI [108], spiral nerve ligation (SNL) [109], and other peripheral nerve injury models. The roles of CXCR3 and its ligands in chronic neuroinflammatory models have been widely studied, and they constitute the focus of CXCR3 research in the field of nervous system diseases.

The transcription factor C/EBP*α* competitively binds to the promoter region of CXCR3 DNA, inhibiting DNMT3b, a crucial enzyme involved in CXCR3 DNA methylation in spinal cord neurons [110]. Following SNL, there is a decrease in DNMT3b expression and an increase in C/EBP*α* expression in spinal cord neurons, leading to demethylation of CXCR3 DNA. This process enhances the transcription and expression of CXCR3, ultimately contributing to the development of neuropathic pain [39].

Previous studies have consistently suggested that the binding of CXCR3 to its ligands promotes neuropathic pain and that intrathecal injections of CXCL4, CXCL10, and CXCL11 can directly induce hyperalgesia in healthy mice [31]. The expression levels of CXCR3 and its ligands increased significantly after CCI and SNL. However, there is still controversy about the specific role of each ligand and the specific mechanism underlying how it promotes pain, as well as about the localization of CXCR3.

Most previous findings support CXCL4 and CXCL9 as key factors in the maintenance of neuropathic pain. CXCL4 was previously not considered to be involved in neurological diseases, but Piotrowska et al. [31] found that CXCL4 in the spinal cord continued to increase within 14 days after CCI, CXCL4 in the DRG continued to be strongly expressed from 7 to 28 days, and CXCL9 also continued to be strongly expressed in the late stage of neuropathic pain. The strong expression of CXCL9 in the late stages of neuropathic pain has also been validated in other experiments [51]. These increases in CXCL4 and CXCL9 combine with CXCR3 to activate microglia in the spinal cord and DRG to prolong neuropathic pain. Notably, activated microglia and macrophages in rats have the ability to release CXCL4 [110], and activated microglia are able to activate more microglia by secreting CXCL4, thereby forming a positive feedback to enhance and maintain neuropathic pain.

CXCL11 plays an early role in neuropathic pain. In a study using a mouse CCI model, Li et al. [51] also detected CXCL9, CXCL10, and CXCL11 and found that CXCL11 expression began to increase significantly at 3 days after surgery and activated CXCR3 in DRG neurons. This result was verified by Piotrowska et al. [31], who found that CXCL11 expression was significantly increased in the spinal cord of rats at 2 days after CCI.

However, different results were obtained by Kong et al. In an SNL model, they found that the CXCL9, CXCL10, and CXCL11 expression levels increased after SNL but at different time points. The expression levels of CXCL9 and CXCL11 increased only in the late stage of SNL, while that of CXCL10 continued to increase in the early stage after SNL and only CXCL10 could enhance the excitability of DRG neurons [52]. The expression levels of CXCL10 and CXCR3 in DRG increased and showed a high degree of co-localization [52]. This phenomenon has also been verified in a cancer pain model of bone metastasis in breast cancer [111]. Thus, CXCL10 may be released and combined with CXCR3 by DRG in an autocrine or paracrine manner after SNL. CXCL10 combined with CXCR3 could activate the p38 and ERK signaling pathways in DRG neurons [52], and p38 could directly phosphorylate Nav1.8 and increase the density of Nav1.8-type currents. This sodium channel has been shown to be associated with neuropathic pain by regulating the action potentials and firing properties of DRG neurons [112]. Moreover, following SNL, astrocytes release CXCL10, which activates CXCR3 in spinal cord neurons, initiating the activation of NMDAR via the ERK/NR2B pathway. This cascade ultimately increases AMPAR expression, thereby amplifying synaptic excitability [43, 44]. The CXCL10/CXCR3 pathway can increase the number of action potentials in DRG neurons, enhance the excitability of DRG neurons, and then induce neuropathic pain. However, Piotrowska et al. suggested that CXCR3 was strongly expressed only in spinal cord neurons, whereas its expression in DRG did not change significantly.

In addition to changes in the excitability of DRG neurons themselves, immune cells also play a role in the regulation of neuropathic pain. Since most immune cells cannot pass freely through the blood–spinal-cord barrier, an increased permeability of this barrier is a prerequisite for the recruitment and infiltration of circulating immune cells into the spinal cord [113]. In an experiment based on a rat CCI model, Cahill et al. [114] found that blocking the CXCL10/CXCR3 signaling pathway by an intrathecal injection of CXCL10 antibody ameliorated the disruption of the blood–spinal-cord barrier induced by CCI, thereby reducing the migration of T cells into the spinal dorsal horn. T-cell infiltration can promote neuropathic pain, and so the CXCL10/CXCR3 pathway can increase blood–brain barrier permeability and enhance T-cell infiltration to promote neuropathic pain. Notably, the secretion of IFN-*γ* by Th1 cells can promote CXCL10 secretion by neurons and glial cells, which would form a positive feedback system that would play a role in neuropathic pain.

In summary, CXCR3 and its ligand ligands play a role in promoting the development of neuropathic pain. The transcription factor C/EBP*α* inhibits DNMT3b, promoting demethylation of CXCR3 DNA in spinal cord neurons following SNL. This demethylation enhances CXCR3 transcription and expression, contributing to neuropathic pain development. CXCR3-related ligands play varying roles in neuropathic pain. CXCL4 and CXCL9 are implicated in the maintenance phase of neuropathic pain, activating microglia in the spinal cord and DRG. CXCL11 shows early upregulation after injury and activates CXCR3 in DRG neurons, contributing to pain sensitization. Astrocytes release CXCL10 following SNL, activating CXCR3 in spinal cord neurons. This activation triggers the ERK/NR2B pathway, leading to increased AMPAR expression and synaptic excitability, ultimately amplifying neuropathic pain.

### 4.4. CXCR3 Pathway in PNS

**Figure 2:**
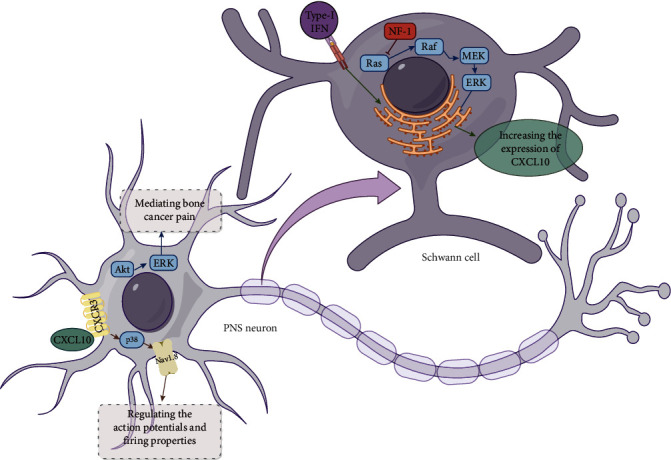
The pathway of CXCR3 and its ligands in PNS cells (by Figdraw). Schwann cells are the main source of CXCR3 ligands in the PNS. Both type-I IFN and the Ras/Raf/MEK/ERK pathways can promote the expression of CXCL10 in Schwann cells. CXCL10 binding to CXCR3 is able to activate p38/Nav1.8 and Akt/ERK pathways in PNS neurons.

In the PNS, the CXCR3 pathway mainly involves Schwann cells and neurons and is shown in Figure 2.

Schwann cells are the main source of CXCR3 ligands in the PNS via two main pathways: (1) similar to glioma, type-I IFN promotes CXCL10 expression in Schwann cells [35], and (2) activation of the Ras/Raf/MEK/ERK pathway also promotes the expression of CXCL10 in Schwann cells [35], and NF-1 as an inhibitor of Ras protein can inhibit this pathway and suppress the expression of CXCL10 in Schwann cells [35].

Unlike neurons in the CNS, it is still controversial whether PNS neurons possess the capacity to secrete CXCL10 on their own. However, several studies have shown that the CXCR3 pathway can play a role in PNS neurons. Neurons in the TME release CXCL10, which interacts with CXCR3 on Nonneurogenic tumor cells, activating downstream pathways (Akt, MEK, RAC) that facilitate tumor infiltration into peripheral nervous tissues. This pathway also mediates cancer pain through Akt and ERK1/2 signaling in spinal cord neurons [107]. On the one hand, CXCL10 binding to CXCR3 is able to activate p38, which in turn activates Nav1.8 [40]; on the other hand, activated CXCR3 could activate the Akt/ERK and MAPK pathways [40, 52], thereby increasing neuronal excitability.

## 5. Conclusions and Future Perspectives

Few studies have investigated CXCR3 and its ligands in nervous system-related diseases, with previous research primarily focusing on tumors, CNS degeneration, and neuropathic pain. In these diseases, the specific binding between CXCR3 and its ligands facilitates communication between neurons and glial cells, among glial cells themselves, and between nerve tissue and immune cells. These interactions play crucial regulatory roles in disease progression by modulating glial cell function and immune responses from immune cells or directly regulating neuronal activity. However, despite CXCR3 being a common inflammatory chemokine receptor, its precise roles in various diseases remain unclear. Controversies surrounding the involvement of the CXCR3 pathway persist in conditions such as glioma [48, 54, 59]; this uncertainty may stem from distinct inflammatory characteristics.

Inflammation possesses dual properties of inducing damage and facilitating repair. Appropriate inflammatory responses can promote tissue repair, while excessive inflammation can lead to detrimental effects such as immune reactions and aberrant cellular transformations during repair processes. Therefore, future research into inflammatory chemokines, including CXCR3, should focus on the intensities of pathway activations and fully explore the role of the CXCR3 pathway in various diseases. Furthermore, since nerve injuries are inevitably accompanied by inflammation, chemokines play a significant role in recruiting immune cells and promoting phenotypic changes within glial cells during nerve regeneration processes. Consequently, characterizing the functional recovery that occurs after nerve injury may represent an important direction for investigating numerous inflammatory chemokines, including CXCR3, in future research.

Frankly, this area of research is still evolving, with most studies focusing on changes in CXCR3 and its ligand expression levels and their correlation with diseases rather than delving deep into downstream pathways. Our comparison of CXCR3-related pathways in the CNS and PNS revealed that the regulation of CXCR3 and its ligands' expression levels varied among different cells. CXCR3 activation primarily exerts its physiological effects via the MAPK pathway. The MAPK signaling pathway is not only the core pathway of CXCR3 after activation but also participates in the expression of CXCR3 ligands in the nervous system, which is the core pathway via which CXCR3 exerts physiological functions. ERK within the MAPK pathway is widely involved in various nervous system diseases and plays key roles across different tissues, cells, and their interactions.

## Figures and Tables

**Table 1:** The pathway and function of CXCR3 and its ligands in the nervous system.

Expression/producer cells	Molecules and pathways	Mechanism	Species	Disease	References
CNS neuron	C/EBP*α*	Inhibiting the DNMT3b to reduce the methylation of CXCR3	Mice	SNL	[39]
CNS neuron	DNMT3b	Maintaining CXCR3 methylation	Mice	SNL	[39]
CNS neuron	ERK	Activated by CXCR3	Mice	SNL	[39]
CNS neuron	Akt/ERK	Activated by CXCR3	Rat	Bone metastasis in breast cancer	[40]
CNS neuron	STAT1 STAT3	Increasing the expression of CXCL10	Mice	Optic nerve crush	[41]
CNS neuron	ERK1/2	Activated by CXCR3	Mice	Alzheimer's disease	[42]
CNS neuron	MEK/ERK/NR2B/NMDAR/AMPAR	Activated by CXCR3	Rat	Inflammatory pain	[43, 44]
Glioma	ERK1/2	Activated by CXCR3	Human	Glioma	[45, 46]
Glioma	Calcium channel	Activated by CXCR3	Human	Glioma	[45]
Glioma	LRP1	Enhancing the internalization of CXCR3	Human	Glioma	[45]
Glioma	Celecoxib/NF-*κ*B	Inhibiting the expression of CXCR3	Mice	Glioma	[47]
Glioma	Poly(I:C)/Type-I IFN/1IFNAR	Increasing the expression of CXCL10	Mice	Glioma	[48]
Astrocyte	TDP-43	Increasing the expression of CXCL9/CXCL10	Mice	Alzheimer's disease	[49]
Astrocyte	IFN-*γ*	Increasing the expression of CXCL11	human	Cell experiment	[27]
Microglia	IFN-*γ*	Increasing the expression of CXCL11	human	Cell experiment	[50]
Microglia	p38/MAPK	Activated by CXCR3	Mice	CCI	[51]
DRG	p38/Nav1.8	Activated by CXCR3	Mice	SNL	[52]
DRG	ERK	Activated by CXCR3	Mice	SNL	[52]
DRG	Akt/ERK	Activated by CXCR3	Rat	Bone metastasis in breast cancer	[40]
Schwann cell	Raf	Increasing the expression of CXCL10	Mice	Nerve injury	[35]
Schwann cell	Ras/Raf/MEK/ERK/AP-1	Increasing the expression of CXCL10	Mice	Neurofibroma	[53]
Schwann cell	NF-1	Inhibiting Ras to reduce the expression of CXCL10	Mice	Neurofibroma	[53]
Schwann cell	Type-I IFN/1FNAR	Increasing the expression of CXCL10	Mice	Neurofibroma	[53]

## Data Availability

Data availability is not applicable to this article as no new data were created or analyzed in this study.
